# A Rare Case of Hypothermia-Induced ST Segment Elevation

**DOI:** 10.7759/cureus.16365

**Published:** 2021-07-13

**Authors:** Arnold N Forlemu, Hursh Sarma, Mohammad Khatib

**Affiliations:** 1 Internal Medicine, Creighton University School of Medicine, Phoenix, USA

**Keywords:** hypothermia, st segment elevation, osborn wave, ekg, differential diagnosis

## Abstract

Prompt recognition of ST segment elevation myocardial infarction (STEMI) is critical as it has significant management and outcome implications, often leading to emergent cardiac catheterization for revascularization. However, other conditions such as pulmonary embolism, myocarditis, hyperkalemia, hypercalcemia, hypothermia, drug overdose, septic shock, left bundle branch block, left ventricular aneurysm, pericarditis, Brugada syndrome, and Takotsubo cardiomyopathy can mimic this presentation on electrocardiography (EKG) and need to be recognized to avoid unnecessary procedures and improve treatment outcomes. We report a case of prominent Osborn waves on EKG from significant hypothermia incorrectly labeled as STEMI. We also provide a literature review on EKG manifestations of hypothermia and the mechanism of those changes, the differential diagnoses of ST segment elevation and their management.

## Introduction

Hypothermia, a low core body temperature <35˚C, can have several electrocardiographic manifestations including Osborn (J) waves, QT prolongation, ST segment changes, and Brugada pattern morphology [1]. Rarely, hypothermia can present as ST segment elevation (STE) mimicking myocardial infarction. It is important to recognize hypothermia in the differential diagnosis of STE. This case report aimed at raising awareness that hypothermia and some other medical conditions can mimic ST elevation myocardial infarction (STEMI). Recognizing these conditions and their electrocardiographic changes can help prevent wrongful diagnosis of STEMI, avoid unnecessary activation of the catheterization laboratory, and improve treatment outcomes. Furthermore, this case report will provide a literature review on the causes of ST segment elevation (STE).

## Case presentation

**Figure 1:**
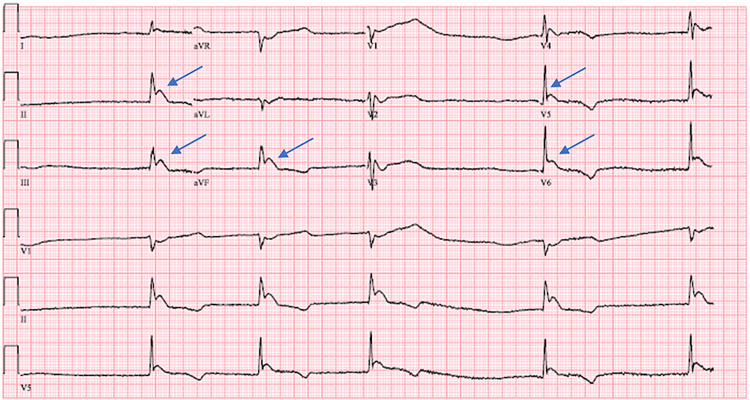
Patient's EKG on admission shows ST elevation inferolateral injury vs acute inferolateral infarct vs Osborn waves (blue arrows), junctional bradycardia (ventricular rate 31 bpm), and non-specific intraventricular conduction delay. The body temperature at the time of EKG was 24˚C. EKG: electrocardiography

A 77-year-old female with an unknown medical history presented to the emergency department (ED) via emergency medical services (EMS) after being found down. No history was available on the presentation. Vital signs in the ED showed temperature at 24˚C, bradycardic at 35 beats per minute, but had elevated blood pressure at 160/72 mmHg. Electrocardiography (EKG) revealed junctional bradycardia with a rate of 31 beats per minute and ST elevation in inferior leads II, III, and augmented vector foot (AVF) and lateral precordial leads v5, v6 (Figure 1).

Laboratory results showed normal troponin level, very low hemoglobin, elevated thyroid-stimulating hormone (TSH), low thyroxine (T4), and low triiodothyronine (T3) (Table 1).

**Table 1:** Pertinent laboratory workup

Parameter	Value	Reference range
Troponin	0.013 ng/ml	<0.34 ng/ml
Hemoglobin	2.4 mg/dl	11.6-14.8 mg/dl
Thyroid-stimulating hormone (TSH)	134 uIU/ml	0.5- 8.9 uIU/ml
Thyroxine (T4)	0.20 ng/dl	0.78-2.19 ng/dl
Triiodothyronine (T3)	1.11 pg/ml	2.77-5.27 pg/ml

**Figure 2:**
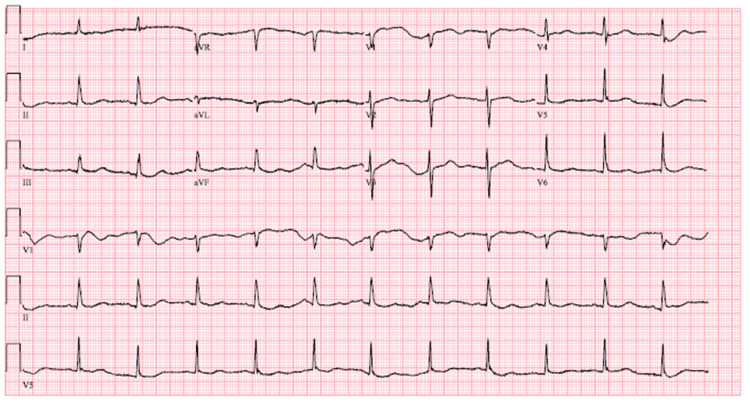
Patient's EKG after rewarming with body temperature now at 36˚C. EKG shows normal sinus rhythm, ventricular rate 70 bpm, prolonged QTc 475, non-specific T wave changes. EKG: electrocardiography

There was a discussion to activate the catheterization lab given EKG findings and altered mental status, however, after initial external rewarming of the patient, she was more awake and alert, and repeat EKG showed resolution of ST changes with normal sinus rhythm and rate at 70 beats per minute (Figure 2).

Her repeat temperature after aggressive rewarming came up to 36˚C. She was transfused three units of packed red blood cells and her hemoglobin improved to 8.2 mg/dl. Per subsequent studies, the anemia was microcytic and hypochromic, iron level was low. The etiology of anemia was likely gastrointestinal, and her hemoglobin remained stable during the hospital stay without any active bleeding. She was started on levothyroxine for her hypothyroidism. It was unclear why she had severe hypothermia in the month of March in Arizona which typically has warm temperatures, but the likely etiology was hypothyroidism. She was eventually discharged with follow-up for gastrointestinal studies.

## Discussion

Hypothermia is a low core body temperature <35˚C [1]. Body temperature is a balance between heat production by cellular metabolism and heat loss via skin and lungs. The hypothalamus response to cold stress by stimulating heat production through shivering, increased thyroid, catecholamine, and adrenal activity. Shelter and clothing are also critical to guard against cold temperatures [2]. Declining body temperatures due to exposure to cold temperatures for a prolonged period or due to failure of the hypothalamus, thyroid, or adrenals in increasing/maintaining body temperatures leads to a decrease in tissue metabolism, ventilation, cardiac output, and neurologic function [2,3]. Causes of hypothermia include prolonged exposure to cold temperatures (winter months), homelessness, hypothyroidism, sepsis, malnutrition, impaired shivering, adrenal insufficiency, hypoglycemia, thiamine deficiency, ethanol abuse, and carbon monoxide intoxication [4]. The patient in this case report was found down and altered during the spring season in Arizona, which typically has warm temperatures. She did not appear septic, had a normal body mass index and blood glucose level, but with profoundly elevated TSH, making hypothyroidism the potential cause of her hypothermia.

EKG manifestations of hypothermia

The presence of STE in a patient with chest pain is usually considered STEMI. However, non-ischemic causes of STE should also be in the differential diagnosis, especially if the symptoms are less typical of STEMI. Hypothermia is a low core body temperature <35˚C that can present with a variety of EKG findings including sinus bradycardia, J waves, atrial fibrillation, interval prolongation of PR, QRS, or QT, along with junctional rhythms, and ST segment depression [1,5,6]. Rarely, as in our case, hypothermia can manifest as ST segment elevation simulating a STEMI.

Mechanism of EKG changes due to hypothermia

The ST segment starts with the J point at the onset of the plateau phase of the action potential [4-6]. Normally this segment is reflected by an isoelectric horizontal line because myocardial cells at this phase have the same membrane potential with no net voltage gradient across the membrane [4]. Any delay or distortion in shape, height, or duration of the action potential due to myocardial injury leads to a voltage gradient between injured and normal myocardial cells. This leads to STE when an epicardial or a transmural lesion occurs [7]. Hypothermia depresses calcium-dependent adenosine triphosphatase (Ca-ATPase) activity by reducing the number of active calcium pump units [8]. Reduction in calcium uptake delays calcium inward current, resulting in myocardial conduction delay that can lead to STEs and arrhythmias. The J or Osborn wave, a convex deflection between the QRS complex and the ST segment, is a reversible wave present in about 80% of patients that are hypothermic [1,9]. Although this wave is very sensitive and very specific for hypothermia, it can be present in other conditions such as myocardial ischemia, hypercalcemia, and intracranial hemorrhage [6,10]. J waves often occur with STE because the J wave can be partially buried in the R wave and represents early repolarization or acidosis occurring in inferolateral leads [10,11].

Other causes of ST elevation

Other conditions can also cause STE and should be considered in the differential. Pulmonary embolism can cause STE in anteroseptal or inferior leads. ECG usually also presents with sinus tachycardia, right bundle branch block, or S1Q3T3 pattern [12]. Hyperkalemia can produce STE that is often downsloping and associated with peaked T waves, flattening P waves, prolonged PR, and widened QRS. Pericarditis often causes diffuse STE with PR segment depression [12]. Brugada syndrome often leads to STE in V1-V3. Other conditions including myocarditis, left bundle branch block, left ventricular aneurysm, Takotsubo cardiomyopathy, hypercalcemia, septic shock, and drug overdose can all cause STE [12]. The patient in this report was not short of breath or septic, had normal potassium levels and her urine drug panel was negative. Hypothermia was a more likely cause in our case and was confirmed by the disappearance of the STE changes after successful rewarming. There were no prior EKGs for comparison in our case, and her troponin levels were normal.

Management of ST elevation

STE management depends on prompt recognition of its cause based on clinical presentation, laboratory, and STE EKG patterns. This step is important because a wrongful diagnosis can potentially lead to worse outcomes. In a patient presenting with hypothermia and STE, airway, breathing, and circulation should be immediately assessed. Prevention of further heat loss and appropriate rewarming should then take place [13]. Rewarming ranges from external rewarming (removing patient’s wet clothing, taking patient into a warm room, applying warm blankets, radiant heat or heating pads) to internal rewarming (intravenous crystalloids, peritoneal and thoracic irrigation). A stepwise approach is preferred beginning with less invasive techniques [13]. In a patient with STE from myocardial infarction, emergent coronary angiography with possible angioplasty or stenting is warranted. Pulmonary embolism-induced STE is managed with anticoagulation. If pericarditis is the cause, anti-inflammatory medications, colchicine or antibiotics may be indicated depending on the cause. Hyperkalemia in this context is managed with calcium gluconate, insulin and glucose, or inhaled albuterol. Myocarditis is often viral related and about 50% of patients recover spontaneously without treatment. Patients presenting with heart failure should be managed with positive inotropes, vasodilators, and diuretics as indicated [12,13].

## Conclusions

Despite documentation of hypothermia causing STE changes, catheterization labs are still sometimes activated for STEMI in these cases. Our case review may help remind providers of the potential for other causes of STE mimicking STEMI. Hypothermia should be considered in the differential of ST elevation found on EKG. Obtaining patient’s presenting symptoms and physical findings starting with vitals is key to the diagnosis, and may prevent unnecessary activation of the catheterization lab. STE should be interpreted in conjunction with clinical presentation as well as other diagnostic tools if available to better narrow the differential diagnosis. A thorough evaluation of hypothermia should be done in all patients presenting with hypothermia with an unknown trigger. In our case, the likely etiology of hypothermia was hypothyroidism and our patient was discharged on levothyroxine. In this case report, we also provided a comprehensive literature review of the differential diagnoses of ST segment elevation on EKG. This may guide providers in their decision-making when they encounter ST segment elevation on EKG.
